# Re-Living Suspense: Emotional and Cognitive Responses During Repeated Exposure to Suspenseful Film

**DOI:** 10.3389/fpsyg.2020.558234

**Published:** 2020-10-23

**Authors:** Changui Chun, Byungho Park, Chungkon Shi

**Affiliations:** ^1^Graduate School of Culture Technology, Korea Advanced Institute of Science and Technology, Daejeon, South Korea; ^2^College of Business, Korea Advanced Institute of Science and Technology, Seoul, South Korea

**Keywords:** suspense, suspenseful film, repeated exposure, reliving, psychophysiology

## Abstract

Arguments about the effects of repeated exposure to a suspenseful narrative raise controversial disputes over the paradox of suspense. The lexical meaning and the theoretical analyses of suspense imply that suspense cannot be experienced repeatedly because, in such cases, the knowledge from prior viewings and the resolution of outcome will eliminate tension and suspense. However, previous studies have argued that suspense can be re-experienced even when the participants know the outcome or repeatedly confront a suspenseful narrative. This study investigated the effects of repeated exposure to a suspenseful film by collecting self-reported questionnaires and measuring psychophysiological responses. The participants (*N* = 50) watched clips of a suspenseful film three times and answered self-reported items regarding suspense, arousal, and enjoyment. Psychophysiological data, including skin conductance level (SCL) and electrocardiogram (ECG), were collected while the participants watched the video clips. It was hypothesized that self-reported suspense, arousal, and enjoyment as well as the physiological indices of arousal (SCL) and attention (ECG) would decrease upon repeated viewing of suspenseful clips. Furthermore, it was postulated that there would be inter-group differences depending on the awareness of potential or definite change in outcome at the end of repeatedly shown suspenseful events. The results showed that self-reported suspense and arousal, as well as the physiological measures of SCL, declined with repeated exposure, although there were no significant differences on self-reported enjoyment. No group difference was found in self-reported items, but meaningful significant changes were observed in the group comparison of SCL and ECG. The findings suggest that repeated exposure to suspenseful films could result in affective habituation or desensitization to repeated stimuli. The implications and the limitations of the current study and suggestions for future research are discussed.

## Introduction

Many people watch suspenseful movies, such as action and thriller films, and read suspenseful books, including detective novels and mysteries, for entertainment purposes; in other words, people commonly consume suspenseful content provided by the entertainment media industry. The *Random House Dictionary* (1987) defines suspense as “a state or condition of mental uncertainty or excitement, as in awaiting a decision or outcome, usually accompanied by a degree of apprehension or anxiety” (p. 1917)^1^. Scholars suggest that suspense is an anxious state of mind that readers or viewers who feel sympathy for protagonists experience while following a series of events with an uncertain outcome (Zillmann, 1991a,b, 1995, 1996; Carroll, 1996; Vorderer, 1996).

Many researchers argue that suspense arises from uncertainty (Chatman, 1980; Walton, 1990; Gerrig, 1993; Carroll, 1996; Zillmann, 1996). Although one of the main factors that generate suspense is uncertainty, according to the lexical meaning of the term and its theoretical conceptualization, uncertainty diminishes upon repeated exposure to a suspenseful narrative. However, it has been proposed that suspense can be experienced repeatedly, even when the uncertainty of outcome is already resolved (Gerrig, 1989, 1996, 1997; Brewer, 1996; Carroll, 1996). If uncertainty is necessary for generating suspense, how can the phenomenon of many moviegoers repeatedly watching the same suspenseful movies be explained?

Skulsky (1980) argued that a suspenseful experience could be repeated by sympathizing with the characters and their state of uncertainty while reading narratives or stories. Another explanation for repeated suspenseful experiences suggests that feigned suspense could be generated even after exposure to a suspenseful story (Walton, 1978). Extending the argument by Skulsky (1980), Gerrig (1989) postulated the idea of “anomalous suspense,” in which people experience suspense even in spite of their knowledge of the outcome. Carroll (1996) presented a theoretical argument that the experience of fictional suspense can be repeated because fiction readers can pretend or imagine that they do not know a story’s outcome. He argued that thoughts and imagination elicit readers’ emotions, and suspense can be evoked by the thought that the readers do not know the outcome, not by the readers’ belief that they do know it.

According to Brewer (1996), the contention of Gerrig (1989) and Carroll (1996) is in line with the idea of the willing suspension of memory. Brewer (1996) accounted for the paradox of suspense from the perspective of memory-related postulations. In particular, he suggested that memory-forgetting (that is, forgetting the content of a story after a certain period of time), memory-capacity limitation (the inability to remember all the details of a story even after having read it), and memory-how (the inability to remember how a story reached its suspenseful moment despite knowing of the suspenseful moment itself) explain the paradox of suspense. By contrast, Yanal (1996) insisted that the repeated experience of suspense is impossible because this phenomenon is inconsistent with the definition of suspense.

The contradiction between the conceptualization of suspense and its actual experience is the so-called paradox of suspense. The following fundamental questions arise from this: can one re-experience suspense in repeated exposure to suspenseful films? If so, what are the cognitive and psychological mechanisms underlying that phenomenon? This study attempted to explore these questions through an empirical experiment to observe and analyze audience members’ self-reported measures and psychophysiological responses during repeated exposure to a suspenseful film clip.

## Literature Review and Hypothesis

Although suspense can arise in daily life, such as job interviews and lottery wins (Lehne and Koelsch, 2015), previous research has focused on fictional suspense because repetitive suspense is common in fictional genres such as films and novels.

Research on the paradox of suspense includes philosophical development (Carroll, 1996) and theoretical studies (Brewer, 1996; Vorderer, 1996). Gerrig’s (1989) experiment investigated the cognitive processing of participants confronted with historical events but who already knew their outcomes. Empirical research on musical tension suggested that musical information processes could be automatic and pre-attentive; thus, in music, tension could be experienced repeatedly upon repeated listening to suspenseful sections of musical pieces (Koelsch et al., 2002; Lehne and Koelsch, 2015).

Prior empirical studies regarding suspense have mainly been conducted using self-report responses (Comisky and Bryant, 1982; Hoeken and van Vliet, 2000; Knobloch et al., 2004; Madrigal et al., 2011). Even though self-report questionnaires are widely used in the study of suspense, they have some disadvantages such as answer distortions (Bolls et al., 2001) and the interference of continuous message processing (Ravaja, 2004). On the other hand, psychophysiological measurement can track continuous responses to media processing without interference and provide huge amounts of data for effective use in temporal analyses of media exposure.

Psychophysiology explores changes in the activities of physiological systems caused by psychological inputs (Turner, 1994). Cacioppo and Tassinary (1990) defined this as “the study of cognitive, emotional, and behavioral phenomena as related to and revealed through physiological principles and events” (p. 3). In this definition, psychophysiology addresses the principal questions about human processes and provides the research equipment that spans the biological, behavioral, and social sciences.

Among various psychophysiological measures, electrodermal activity (EDA) and heart rate (HR) are frequently employed as indices of cognitive and emotional processing (Ravaja, 2004). Skin conductance (SC), a representative measure of EDA, is regarded as a direct index of arousal. SC has been widely used in media research to analyze psychophysiological responses and arousal (Lang et al., 1999, 2005a, 2007; Bolls et al., 2001; Ravaja et al., 2006; Lim and Reeves, 2009).

The measure of SC is closely related to an individual’s subjective emotional intensity (Ravaja, 2004). Therefore, the intensity of arousal to suspenseful media exposure can be used to measure audience members’ experience of suspense. Unlike SC, HR is under the dual control of the sympathetic nervous system and the parasympathetic nervous system (Potter and Bolls, 2012). Thus, it is difficult to interpret the meaning of HR changes precisely. In media psychology research, which mostly limits subjects’ physical activity, an increase in HR has been construed to represent internal cognitive activity, emotional arousal, and positive emotional responses. Meanwhile, a decrease in HR can be interpreted as the acquisition of information (e.g., TV programs), attention, and the experience of negative emotions (Lang et al., 1995; Bolls et al., 2001; Bradley et al., 2001; Ravaja, 2004).

Recently, many studies have been increasingly using psychophysiological measures in various research fields. In one experiment, psychophysiological responses were examined among young video game players who experienced different game events (Ravaja et al., 2006). Lim and Reeves (2009) investigated the effects of avatar in virtual reality chosen by player and the visual perspective (i.e., first-person vs. third-person point of view) by measuring physiological arousal. Along with video game research, in media experiments, psychophysiological measures have been particularly useful for investigating audience responses to visual stimuli, as audiovisual materials such as films, movies, and television programs are composed of temporal visual sequences and include continuous music and sound. Lang et al. (1999) observed TV viewers’ HR and SC to investigate how the arousing content and pace of the television message affect information processing. Furthermore, audio listeners’ psychophysiological responses have been collected and analyzed in the same context (Potter, 2000; Bolls and Lang, 2003). Suckfull (2000) examined the psychophysiological effects of narrative structures on film viewers in relation to the moment of an impact and the protagonist. HR changes were analyzed to observe the attentional processes of film viewers, and the results demonstrated how narrative structures linked to the protagonist could evoke significant reactions.

There have been several psychophysiological research studies on suspenseful narrative experience. However, such measures can be useful for observing responses to repeated exposures to suspenseful film stimuli, as EDA and HR are frequently employed to observe emotional arousal (Ravaja, 2004). Moreover, a suspenseful experience is a constant emotional response and would fit psychophysiological evaluation well because the approach can obtain and use a large number of data points from audience reactions to continuous media exposure.

Zillmann et al. (1975) measured children’s HR and skin temperature as they viewed a suspenseful adventure story including narration and pictures, presented *via* videotape on television. Their HR increased with incremental degrees of suspense, but there was no corresponding significant change in skin temperature. It was also found that both HR and skin temperature dropped upon the resolution of the suspense.

Hubert and de Jong-Meyer (1991) investigated emotional responses to a suspenseful film. They chose scenes from two films to evoke different affective conditions: a pleasant cartoon film and a suspense film with repulsive creatures such as snakes and spiders. They then measured autonomic activity including HR, SCL, and endocrine responses. They found that the segment from the suspense film elicited an increase in SCL and a temporary decrease in HR, whereas the cartoon film induced a temporary decrease in HR and a rapid decline in SCL.

The psychophysiological and subjective impact of an emotional movie has also been explored in previous studies (Rottenberg et al., 2007; Codispoti et al., 2008; Carvalho et al., 2012). Codispoti et al. (2008) revealed that highly arousing emotional film clips, regardless of their valence, provoke orienting, and sustained attention as reflected by HR deceleration and larger SCL. Similarly, in a study to develop an emotional movie database, the results demonstrated that sustained exposure to emotional movie clips, including horror and erotic film, elicited a pattern of SCL increase and HR deceleration (Carvalho et al., 2012).

A functional magnetic resonance imaging (fMRI) study was conducted to observe the neural activity of readers of suspenseful stories (Lehne et al., 2015). fMRI data were acquired while the participants read a suspenseful literary text. The brain areas activated while reading were related to social cognition and predictive inference. This suggests that the cognitive and the emotional processing of reading suspenseful text may include components of identification with characters and the expectation of what will happen next in the narrative. Another fMRI study which investigated the brain dynamics of audiences watching a suspenseful movie revealed that the brain regions, during exposure to a suspenseful moment of a suspense film, were associated with emotional salience and higher cognitive processes (Schmälzle and Grall, 2020). This result indicated that the neural activity of viewing a suspenseful film could be related to the motivated attention and psychological tension.

### Suspense and Arousal

This study examined the effect of repeated exposure to an identical suspenseful clip of a suspense film by observing self-reported suspense and arousal as well as physiological indices of arousal (SCL) and attention (ECG). In addition, using the unique and tricky structure of a selected film material that combines repeated chase scenes which have little difference, followed by three different results, the experiment was designed to recreate a viewing experience similar to that of the actual film, that is, the participants were expected to watch the experimental material with a narrative structure similar to that of the original film.

There are few, if any, empirical studies regarding repeated exposure to suspenseful narratives, particularly for audiovisual stimuli. Brewer’s (1996) empirical experiment provided evidence that rereading suspenseful stories (texts) elicited an overall reduction of suspense level, but not to the baseline measured in a non-suspenseful narrative. Other theorists likewise generally agree that the repeated experience of suspenseful stories decreases the degree of suspense. Gerrig (1989); Carroll (1996), and Hoeken and van Vliet (2000) argued that suspense can be repeated even when the reader or viewer knows the outcome of events, but these claims do not include the effect of the whole repeated experience of suspense appreciation.

A study that explored the effect of repeated exposure to violent media experiences showed that the psychological impact lessened upon repeated experience due to a desensitization effect (Fanti et al., 2009). Moreover, it was shown that repeated exposure to emotional pictures results with affective habituation (Codispoti et al., 2006, 2016; Ferrari et al., 2020). In particular, SC and HR habituated rapidly with repetitive presentations of emotional pictures (Codispoti et al., 2006). Repeated exposure to a suspenseful narrative can likewise be expected to reduce the audience’s arousal. It has been previously reported that arousing content elicited both self-reported arousal and physiological responses (Lang et al., 1999; Reeves et al., 1999; Sundar and Kalyanaraman, 2004; Park and Bailey, 2017). Hubert and de Jong-Meyer’s (1991) experiment revealed that suspense films elicited more arousal than calm video clips.

Based on the perspectives derived from previous studies, this study hypothesized:

*Hypothesis 1:* Repeated exposure to a suspenseful film will decrease the degree of suspense.
*H1a:* Repeated exposure to a suspenseful film will decrease self-reported suspense and arousal.
*H1b:* Repeated exposure to a suspenseful film will decrease SCL.

### Attention

In Bezdek and Gerrig’s (2017) study, a suspenseful narrative captured the audience’s attention, resulting in more cognitive resources allocated to the attention process. In addition, a previous research study revealed that danger increased media users’ attention (Lang et al., 2005b). Therefore, suspense accompanying threat toward the protagonist attracts the audience’s attention. Consequently, as the degree of suspense decreases upon repeated exposure, attention can also decrease as was shown in previous studies regarding diminished attention for repeated exposure to emotional stimuli (Codispoti et al., 2016; Ferrari et al., 2020). When attention to the stimulus decreases, cognitive resources allocated primarily to encoding decrease, which can be observed by the increase in HR (Bolls et al., 2001; Lang et al., 2005b). From this perspective, the following was hypothesized:

*Hypothesis 2:* In repeated exposure to a suspenseful film, cardiac acceleration (increase in HR), which represents a decrease in the attention level, will be observed.

### Enjoyment

How audiences enjoy media experiences depends on factors such as immersion, attention, and arousal, with a higher level of these factors eliciting more enjoyment (Brewer and Lichtenstein, 1982; Hoeken and van Vliet, 2000; Busselle and Bilandzic, 2009; Madrigal et al., 2011). Madrigal et al. (2011) found that relief experienced at resolving a tension was a crucial mediating factor in a suspense narrative experience. In our study, the participants watched three different video clips that included repeated suspenseful scenes. The expectation that a decrease in arousal and attention would lower the audience’s enjoyment led to the following hypothesis:

*Hypothesis 3:* Repeated exposure to a suspenseful film will result in a decrease of self-reported enjoyment.

### Group Difference in Prediction

The cognitive process of suspenseful experience includes prediction and anticipation of the result that follows the suspenseful events (Tan and Diteweg, 1996; Lehne et al., 2015). In repeated exposure to a suspenseful narrative, suspense diminishes if the repetitive stories are identical to previous ones that are stored in the viewers’ memory. In other words, suspense declines when unfolding events coincide with the audience’s prediction and anticipation.

The effect of prediction and anticipation also changes depending on the potential for change in the expected event. Two groups whose awareness about a potential change in the following events differed were compared to investigate the impact of viewers’ cognition on repeated exposure to a suspenseful film. The group that had been informed that the result to follow would definitely change in the subsequent screening of the suspenseful event was expected to experience a different emotional and cognitive processing from the other group that was notified that the result may or may not change. This is due to the effect of uncertainty on the cognition of different result possibilities. According to this assumption, the following was hypothesized:

*Hypothesis 4:* During repeated exposure to a suspenseful film, the self-reported suspense and arousal as well as SCL and ECG of the second subject group that was notified about the definite result change will be different from those of the first subject group that was notified about a possibility of change in the result.

## Materials and Methods

This experiment used a 2 (group: certainty and uncertainty on an upcoming event result) × 3 (repeated viewings of a suspense clip) factorial design. A suspenseful film was chosen among candidate pieces, and the experimental materials of 240-s (4-min) clips were created by editing the film’s scenes. Physiological data were measured while the participants were watching the suspenseful clips, and they answered self-reported items between each viewing of the clips.

### Stimuli

After reviewing several candidates that adopted a repetitive structure of storytelling, including films such as *Edge of Tomorrow* (2014) and *Groundhog Day* (1993), the German film *Run Lola Run* (1998) directed by Tom Tykwer was selected for the experiment. This film was chosen because it was composed of repeated suspenseful sequences that could be effectively edited to meet the purposes of the research questions and hypotheses. Moreover, as it was produced by an independent filmmaker (i.e., non-Hollywood-based studio), the subjects (Koreans) of this study had not been exposed to this particular film. Before being assigned to an experiment, no participant reported a prior experience of watching the film.

*Run Lola Run* is unique in that the event structure consists of three repetitive parts. In each, the female protagonist Lola runs to rescue her lover Mani who belongs to a mafia organization and is being threatened by his boss. The repetitive sequences are divided into a first half, which depicts Lola’s run to Mani, and a second half, which describes the outcome after Lola meets Mani. The three sequences have similar patterns of events and characters but are not identical. In Lola’s first run, she fails to rescue her lover. She then tries to outsmart herself in the second trial, which leads to a difference from the first run, but she is still unsuccessful. In the third trial, she tries to reach Mani in a more effective way, which ultimately changes the outcome (creating a happy ending).

The material was edited from sequences in the original film and was made up of five clips: the prolog (P) (60 s), which explained the background of the story before Lola runs; Lola’s repetitive running sequence (R) (120 s) to be repeated during the experiment, the first outcome with Lola’s death (O1) (120 s), the second outcome with Mani’s death (O2) (120 s), and the third outcome, in which the crisis is resolved (O3) (120 s). Each clip except for the prolog was produced by mixing the film sequences and was edited to equal 120 s for psychophysiological data comparison. The repetitive part (R) of Lola running was used to observe the effect of repeated exposure to a suspenseful film. The three outcomes following the R-clip were created to investigate whether the viewers’ responses would differ when the result changed. Each outcome clip (O1, O2, and O3) was attached to the repetitive part (R-clip), resulting in three stimuli (R + O1, R + O2, and R + O3). The material clips were edited with full-HD (1,920 × 1,080) resolution and 30 fps (frames per second).

### Dependent Measures

#### Self-Reported Measures

##### Suspense

This study observed the viewers’ suspense only for the repeated clips (R), not for the whole exposure which includes each session’s outcomes. Therefore, a single-item, nine-point Likert scale question was used to measure suspense: “How much suspense did you feel while watching the scene in which Lola is running?” (1 = very little, 9 = very much).

##### Arousal

The participants were asked a question to measure arousal: “How aroused were you while watching the scene in which Lola is running?” With the item, a nine-point scale Self-Assessment Manikin (SAM) pictogram was presented as a choice. SAM (Bradley and Lang, 1994) is a set of pictograms used to observe media users’ emotional responses, such as arousal, presence, and positive/negative valence. In the actual employment of SAM, the participants select one of nine levels–pictogram items–that most closely describes their emotional state (level 1 = very little, level 9 = very much).

##### Enjoyment

To describe their enjoyment, the participants chose a numeral on a nine-point Likert scale in response to the following item: “How much enjoyment did you feel while watching the clip?” The possible responses ranged from 1 (very little) to 9 (very much).

#### Physiological Measures

##### SCL

The participants’ SCL was measured as an index of their physiological arousal. SCL has been used as a direct index of arousal in various experiments. Arousal activates the sympathetic nervous system and increases SCL (Potter and Bolls, 2012). In the experiment, psychophysiological measures, including SCL and ECG, were gathered using the biofeedback machine MP-150 produced by Biopac Systems Inc. Two Ag–AgCL electrodes, the components of SS3LA transducers manufactured by Biopac, Inc., were attached to the index and the ring fingers of the participants’ left hand to measure SCL. The dataset recorded at 1,000 Hz frequency was down-sampled to 50 Hz and averaged to the second for statistical analysis by using *AcqKnowledge* software, a data processing program bundled with MP-150 systems. Each average value per second was reduced by the gap between the original value and the baseline that was measured while the participants watched a blank screen for 5 s before receiving the stimulation; this is because SCL tends to decrease during measurement.

The participants of the current experiment were supposed to watch the suspenseful video while sitting in a chair; under this condition, SCL decreased continuously even though suspenseful clips elicit arousal since sweat evaporates more than in perspiration. Therefore, the baseline was modified in each measurement to ensure the experiments’ reliability. Measuring relatively different psychophysiological values has been used in various media research studies (Bradley and Lang, 2000; Grabe et al., 2000; Bradley et al., 2001).

##### ECG (HR)

The allocated cognitive resources increase when media users pay more attention, and this can be identified by phasic HR deceleration (Bradley, 2009). Previous studies have likewise found that attention can be measured by the decrease of HR (Bolls et al., 2001; Lang et al., 2005b).

In the current experiment, ECG data were collected to observe the participants’ attention. Three Ag–AgCl electrodes connected to a Biopac 150 unit were attached to the inner elbows of the participants’ arms and to the left inner wrist to measure ECG signals. Data recorded at 1,000 Hz were down-sampled and converted to the inter-beat interval (IBI) and averaged to the second by *AcqKnowledge* software. IBI is a measured value of the gap between heartbeats and has an inverse relationship with HR (i.e., a higher IBI means a lower HR and *vice versa*). Therefore, the degree of attention is proportional to the IBI level. As with the SCL data, the average IBI value per second was calculated by the difference between the original value and the baseline, as measured while the participants watched the blank screen for 5 s.

### Participants

A total of 50 graduate and undergraduate students from two large universities located in Seoul, Korea were recruited and received monetary payment of KRW 20,000 (about US $18) for their participation. The participants consisted of 22 females and 28 males, and their mean age was 26.4 years (*SD* = 4.15).

### Procedure

**FIGURE 1:**
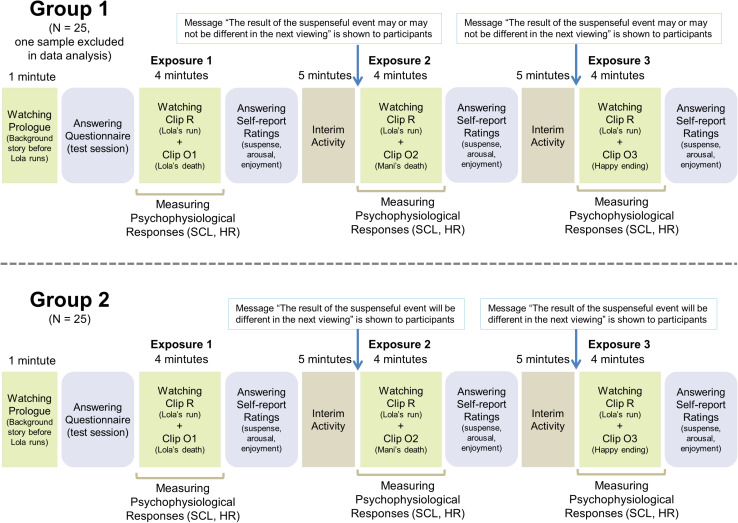
Experiment’s procedure.

After the participants consented to participation, they were given instructions about the experiment’s procedure. They were seated in front of the computer systems and were told to watch the 24-in. LCD monitor carefully as the film was presented. They were also requested to choose the proper items by clicking the mouse when the questions were given. The 50 participants were randomly assigned to group 1 and group 2 equally (25 subjects for each group): group 1 consisted of 12 females and 13 males, and their mean age was 27.9 years (*SD* = 4.43); group 2 was comprised of 10 females and 15 males, and their mean age was 25.0 years (*SD* = 3.32). The sensors to measure SCL and ECG were attached to the participants’ fingers, wrist, and arm. The subjects were then shown the prolog clip and given a practice test session of filling in a questionnaire. After the session, they watched the first suspense clip (R+O1) and answered the questions measuring self-reported ratings. This process was conducted for the second exposure (R+O2) and third exposure (R+O3). Before the second and the third exposures, there were interim activities wherein the participants listened to audio clips and filled in questionnaires. In addition, before the second and the third clips were presented, the participants of each group were notified of different messages. Group 1 was given the message “The result of the suspenseful event may or may not be different in the next viewing,” while group 2 was given the message “The result of the suspenseful event will be different in the next viewing.” While the participants watched each clip, the equipment gathered psychophysiological responses. MediaLab 2008, which is a computerized empirical research software program made by Empirisoft, was used in this experiment. The whole process, shown in Figure 1, was conducted *via* computerized tests, wherein the participants watched experimental material, read instructions, and clicked questionnaire items on the computer monitor.

### Data Analysis

For self-reported data, a 2 (group: certainty and uncertainty) × 3 (repeated viewing: exposure 1, exposure 2, and exposure 3) repeated-measures ANOVA was conducted by using IBM SPSS Statistics (version 22) to verify the hypotheses. All physiological data for the 240 s, including SCL and IBI, were averaged to seconds after deducting the baseline difference, as indicated in section “Physiological Measures,” resulting in 240 data points for each exposure. A 2 (group) × 3 (repeated viewing) repeated-measures ANOVA analysis was conducted for the repeated parts of the clips, which lasted for the first half (120 s) of each exposure (240 s), as statistical verification is needed for the repeated exposure.

When conducting data analysis, one participant’s data in group 1 was excluded due to technical difficulties that led to incorrect data collection (resulting *n* = 49 for data analysis). Statistical analysis was conducted to explore within-subject effect (*n* = 49) for H1a, H1b, H2, and H3 and the between-subjects effect (group 1 subjects = 24, group 2 subjects = 25) for H4, which required a group comparison. All main effects and interactions of the repeated-measures ANOVA are shown in Table 1.

**TABLE 1:** Main effects and interactions of repeated-measures ANOVA.

	*F*	*df*	*p*
**Self-reported ratings (within-subject)**			
Self-reported suspense	20.21	2, 98	<0.001*
Self-reported arousal	13.54	2, 98	<0.001*
Self-reported enjoyment	2.39	2, 98	0.097 (ns)
**Self-reported ratings (between-subjects)**			
Self-reported suspense × group	2.16	2, 94	0.122 (ns)
Self-reported arousal × group	1.81	2, 94	0.170 (ns)
**Skin conductance level for the repeated parts**			
**(the first 120 s; within-subjects)**			
Exposure	4.08	2, 96	0.020**
Time	5.06	119, 5,712	<0.001*
Exposure × time	1.24	238, 11,424	0.007**
**Skin conductance level for the repeated parts**			
**(the first 120 s; between-subjects)**			
Exposure × group	2.2	2, 94	0.117 (ns)
Group × time	1.25	119, 5,593	0.034**
Exposure × time × group	0.94	238, 11,186	0.733 (ns)
Group	5.96	1, 47	0.018*
**IBI change for the repeated parts (the first 120 s; within-subjects)**			
Exposure	0.38	2, 96	0.687 (ns)
Time	1.29	119, 5,712	0.019**
Exposure × time	1.15	238, 11,424	0.061 (as)
**IBI change for the repeated parts (the first 120 s; between-subjects)**			
Exposure × group	1.08	2, 94	0.343 (ns)
Group × time	1.09	119, 5,593	0.236 (ns)
Exposure × time × group	1.05	238, 11,186	0.277 (ns)
Group	3.74	1, 47	0.059 (as)

*, significant at <0.001; **, significant at <0.05; ns, not significant; as, approaching significance.

## Results

**FIGURE 2:**
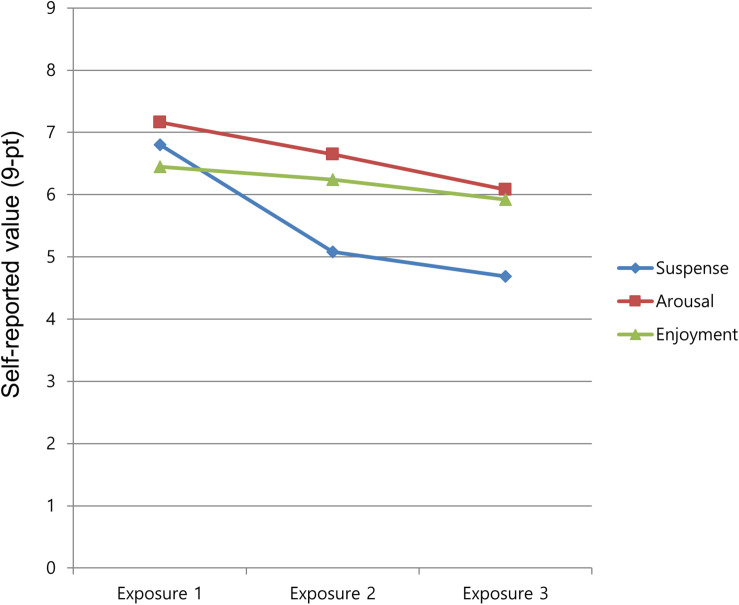
Self-reported ratings on repeated exposure.

There were statistically significant differences in self-reported suspense and arousal. There was a significant main effect of repeated exposure on suspense [*F*(2,98) = 20.21, *p* < 0.001, partial η^2^ = 0.292] and on arousal [*F*(2,98) = 13.54, *p* < 0.001, partial η^2^ = 0.216], as shown in Table 1. A *post hoc* test for self-reported suspense and arousal was conducted. The pairwise comparison of self-reported suspense indicated that there was a significant difference between the first and the second exposures (*p* < 0.001, *M*_exposure1_ = 6.80 vs. *M*_exposure2_ = 5.08), but the difference between the second and third exposures was not statistically significant (*p* = 0.239, *M*_exposure2_ = 5.08 vs. *M*_exposure3_ = 4.69). The pairwise comparison of self-reported arousal revealed that there was a significant difference between the first and the second exposures (*p* = 0.015, *M*_exposure1_ = 7.16 vs. *M*_exposure2_ = 6.65) and the second and the third exposures (*p* < 0.001, *M*_exposure2_ = 6.65 vs. *M*_exposure3_ = 6.08). Therefore, H1a, which predicted that repeated exposure to suspenseful film viewing would decrease self-reported suspense and arousal, was supported. In particular, self-reported suspense and arousal dropped upon the second exposure compared to the first viewing, while the decrease of suspense was not significant in the third exposure, as shown in Figure 2. However, no significant effect was found for self-reported enjoyment. The effect of repeated exposure on enjoyment barely approached significance [*F*(2,98) = 2.39, *p* = 0.097, partial η^2^ = 0.046]. Thus, H3, which predicted that self-reported enjoyment would be lower upon repeated exposure to a suspenseful film, was not supported.

**FIGURE 3:**
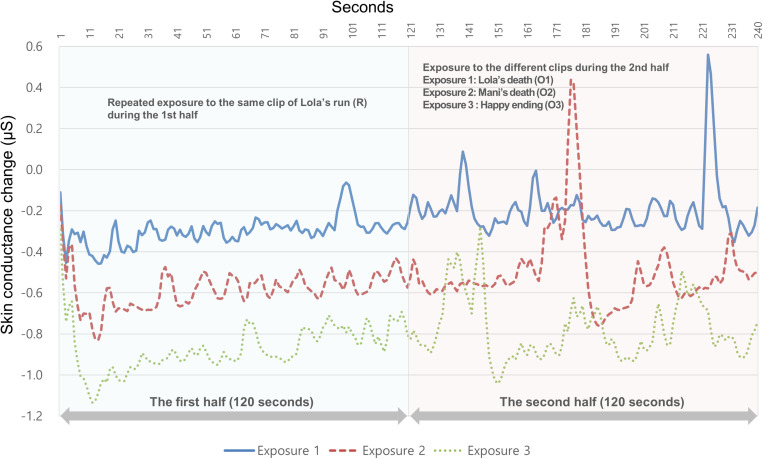
Skin conductance level change × time (s) on repeated exposure.

H1b predicted that SCL, which was measured by the physiological index of arousal, would decrease upon repeated exposure to a suspenseful clip. This was supported in the statistical analysis. The SCL data during the first half of the 120 s that the participants watched three times were statistically analyzed using repeated-measures ANOVA. The within-subject results showed a significant effect for exposure [*F*(2,96) = 4.08, *p* = 0.020, partial η^2^ = 0.078], a significant effect for time [*F*(119,5,712) = 5.06, *p* < 0.001, partial η^2^ = 0.095], and a significant effect for “exposure × time” [*F*(238,11,424) = 1.24, *p* = 0.007, partial η^2^ = 0.025; as shown in Table 1]. The results indicated that the periodic SCL pattern differed over time for each exposure. These are also shown in the mean values of SCL change in each exposure (*M*_exposure1_ = −0.300, *M*_exposure2_ = −0.582, *M*_exposure3_ = −0.869). The profile plot of the three exposures during the overall clip watching is shown in Figure 3. The audience was repeatedly exposed to the same clip of Lola’s run during the first half. The clips that the viewers watched in the second half differed by each exposure. As a result, the graphs of the first half show similar patterns, but the profile tends to go downward as the exposure is repeated, indicating a decrease in SCL. However, the profile of the second half does not show a similarity because of the altered responses to the completely different clips. Overall, the SCL profile plot shows a gradual increase over time. Ravaja (2004) pointed out that psychophysiological responses tend to habituate over relatively long stimulus exposure durations. Despite the habituating pattern of SCL decline that is generally observed in experiments, the tendency toward SCL incline in this study indicates that arousal was increased as the participants watched suspenseful clips.

H2 predicted that HR, a physiological index of attention, would increase for repeated exposure. Repeated-measures ANOVA was conducted for IBI change, which negatively correlates with HR, during the repeated part (the first 120 s). The result showed that the interaction of “exposure × time” approached significance, *F*(238,11,424) = 1.15, *p* = 0.061, partial η^2^ = 0.023. The IBI during the second viewing was higher (HR deceleration) than that of the first viewing (*M*_exposure1_ = 0.040 vs. *M*_exposure2_ = 0.052); however, during the third exposure, it dropped lower (HR acceleration) than the value of the first viewing (*M*_exposure3_ = 0.031 vs. *M*_exposure1_ = 0.040), as shown in Table 2. Therefore, H2 was not clearly supported.

**TABLE 2:** Mean values of dependent variables.

	1st exposure mean (SD)	2nd exposure mean (SD)	3rd exposure mean (SD)
**Psychophysiological measures for the repeated parts (the first 120 s)**			
SCL change (μS) (all subjects)	−0.300 (0.069)	−0.582 (0.092)	−0.869 (0.118)
SCL change (μS) (group 1)	−0.455 (0.109)	−0.837 (0.105)	−1.429 (0.158)
SCL change (μS) (group 2)	−0.150 (0.058)	−0.337 (0.117)	−0.332 (0.104)
IBI change (s) (all subjects)	0.040 (0.025)	0.052 (0.019)	0.031 (0.023)
IBI change (s) (group 1)	0.018 (0.028)	0.012 (0.027)	0.027 (0.028)
IBI change (s) (group 2)	0.062 (0.040)	0.091 (0.028)	0.035 (0.031)
**Self-reported ratings (all nine-point scale)**			
Self-reported suspense (all subjects)	6.80 (1.59)	5.08 (2.22)	4.69 (2.08)
Self-reported suspense (group 1)	7.21 (1.50)	4.88 (2.31)	5.17 (2.20)
Self-reported suspense (group 2)	6.40 (1.63)	5.28 (2.21)	4.24 (1.94)
Self-reported arousal (all subjects)	7.16 (1.52)	6.65 (1.67)	6.08 (1.68)
Self-reported arousal (group 1)	7.50 (1.50)	6.58 (1.50)	6.25 (1.85)
Self-reported arousal (group 2)	6.84 (1.52)	6.72 (1.88)	5.92 (1.55)
Self-reported enjoyment (all subjects)	6.45 (1.94)	6.24 (1.52)	5.92 (1.54)

The between-subjects effects for self-reported suspense and arousal as well as for physiological data including SCL and IBI were examined to investigate H4, which predicted group differences for those measures. The results showed that there were no significant group differences for self-reported suspense nor for self-reported arousal. Meaningful group differences were found in physiological arousal (SCL) through the interaction with time [group × time; *F*(119,5593) = 1.25, *p* = 0.034, partial η^2^ = 0.026] as well as the between-subjects effect [*F*(1,47) = 5.96, *p* = 0.018, partial η^2^ = 0.112]. For IBI, only the between-subjects effect of the whole group comparison approached significance [*F*(1,47) = 3.74, *p* = 0.059, partial η^2^ = 0.074], while other effects were not significant. Therefore, H4 was partly supported.

## Discussion

This study examined the psychological impact of repeated exposure to a suspenseful film by collecting psychophysiological responses as well as self-reported responses such as suspense, arousal, and enjoyment to explore the suspense paradox. The findings provided statistical support for the first hypothesis (H1), which predicted that the degree of suspense as measured by self-reported suspense and arousal and the psychophysiological index of SCL would decrease in repeated exposure to a suspenseful film. All three measures recorded a consistent decline in the second and the third exposures. Specifically, both self-reported arousal and physiological arousal (indexed by SCL) have decreased. These two measures represent the degree of suspense experienced; therefore, the findings support the hypothesis that the viewers’ suspense diminished upon repeated viewing. The outcome is in line with the results of Brewer’s (1996) experiment, which examined suspense text rereading, studies on affective habituation (Codispoti et al., 2006, 2016; Ferrari et al., 2020), and research on desensitization by Fanti et al. (2009). This implies that the primary effect of repeated exposure to suspenseful stimuli is the decrease of suspense and arousal. Nevertheless, the conclusion that viewers of suspenseful films in repetition do not feel suspense or that suspense completely vanishes in repeated watching seems to be implausible because the score for self-reported suspense upon the third exposure was 4.7 (neutral 4) and the arousal score was 6.1, which was comparably high (Table 2). Thus, it can be concluded that while repeated exposure to a suspenseful film reduces the degree of suspense to an extent, it does not entirely eliminate suspense.

The prediction that enjoyment would decrease in repeated exposure was not clearly supported. However, given that the statistical result was approaching significance, the non-significant decrease in self-reported enjoyment over repetition should be taken with caution. It seems that enjoyment level holds over repeated exposure to materials that include identical sequences, and part of the reason may be the introduction of different final outcomes for all three sequences. This is more or less the case for commercial movies that include repetitive sequences followed by different outcomes, such as *Edge of Tomorrow* (2014) and *Groundhog Day* (1993). If the stimulus (the whole video clip consisting of the repeated part and the changing events) used for repeated exposure had been identical for all three viewings, it is highly likely that the desensitization to the stimulus would have bored the subjects more easily, leading to a faster drop in enjoyment level every time it was repeated.

**FIGURE 4:**
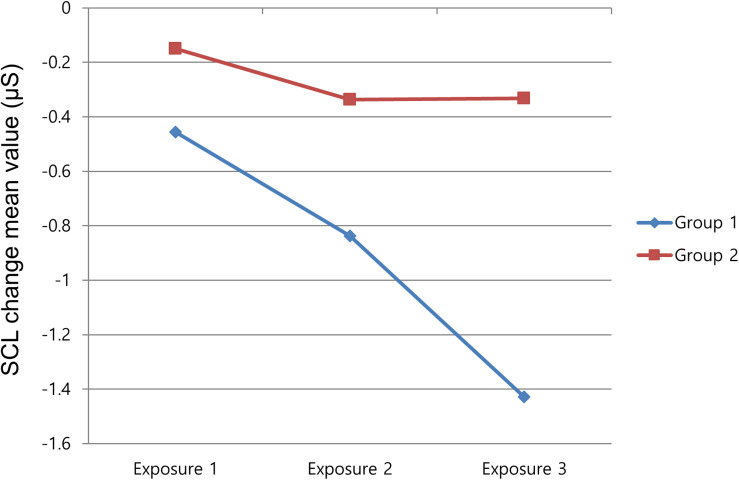
Mean value of skin conductance level change on repeated exposure.

**FIGURE 5:**
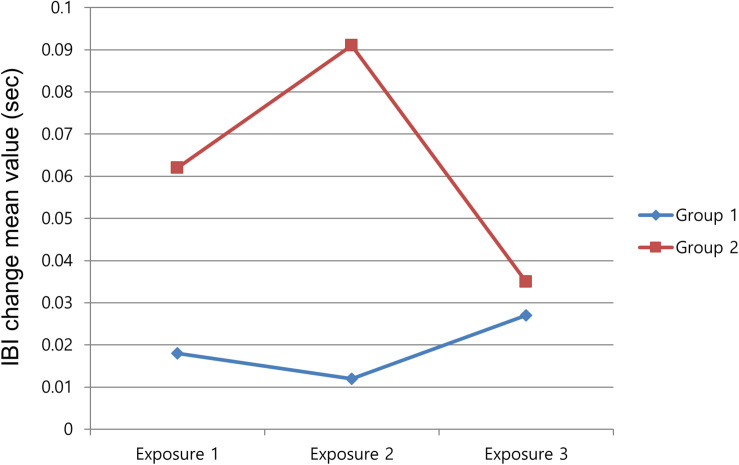
Mean value of inter-beat interval change on repeated exposure.

For the fourth hypothesis, no group difference was found for self-reported suspense and arousal between those informed of the mere possibility of result changes and those who could be certain of the change in event result. Only the whole-group comparisons for SCL and IBI were meaningful. This meant that there were differences between the overall 120 s-averaged responses of SCL and IBI for each group. As shown in Figure 4, SCL in the first group rapidly dropped in the second and the third exposures. On the contrary, SCL in group 2 declined somewhat in the second exposure but remained at almost the same level in the third exposure. The second group was notified of the definite change of the following event, and this seemed to generate an uncertainty of outcomes, which led to the higher level of arousal. In the investigation of IBI, a proportional physiological index of attention, the mean value of the first group was decreased in the second watching and increased in the third watching, as shown in Figure 5. Considering that the first group was notified of the possible change in result after the suspenseful repeated event, the participants might have paid less attention during the second exposure. However, their attention increased in the third viewing after confirming that the results changed in the second viewing. In contrast, the second group, which was notified of the definite change in result, seemed to pay more attention in the second viewing, but their attention dropped in the third viewing after the participants had confirmed that the results changed. These findings imply that different notifications about possible event changes might have a complicated effect on viewers’ responses rather than a single expected effect.

This study provides empirical data, including physiological responses, for repeated exposure to a suspenseful film. One of the advantages that psychophysiological approach can provide is that the continuous process of media exposure can be investigated and partly analyzed. For instance, we could easily separate viewers’ physiological responses into the first half (120 s) and the second half (120 s), although a tricky question was invented to observe viewers’ self-reported suspense and arousal only for the first half of the exposure. Besides that, psychophysiological measurement is based on the autonomic responses of the human body, which can be regarded as more objective than self-reports. The current research presented the same result for the physiological indices and the self-reported items. Thus, the declining tendency of responses related to suspense during repeated exposure was strongly supported by both the subjective and the objective measures. Nevertheless, the result itself may have not come up with the proof needed to explain the paradox of suspense, that is, the outcome of the current study is not sufficient to explain the mechanism underlying the paradox of suspense phenomenon completely. General media re-experiences such as rereading books or watching films repeatedly can occasionally occur with time gaps of days, months, or years. In such cases, the primary factors that determine the impact of repeated exposure are memory effects. The greater the time gap between the initial exposure and the repeated experience for the readers or watchers, the greater the suspense will be, as the time gap is likely to lead media consumers to forget the content, at least in part. From this perspective, future research could focus on how memory effects influence the experience of repeated exposure to suspenseful films. The literature on memory retention generally suggests that forgetting occurs with a power law function (Wixted and Ebbesen, 1991); in other words, memory retention declines sharply over time. Future studies could likewise explore memory effects in repeated exposure to a suspenseful film as mediated by varying time gaps (10 min, 1 h, 1 day, 1 week, etc.).

One of the limitations of the current study is that the participants only watched repeated clips of negative valence. Because the event in the first exposure depicts Lola’s accidental death and the second exposure event reveals the death of her lover, audience members are expected to experience negative valence during the second and the third repeated exposures. If the happy ending were presented in the first exposure, the effect of repeated clip watching might be different, as the audience would expect to experience the positive valence again. As Smith et al. (2006) demonstrated, humans direct more of their attention toward negative information than positive information. Their empirical analysis indicates that watching a clip of negative valence repeatedly leads audiences to devote more attention to negative valence than positive valence. Considering the assumption, future research could focus on the effect of positive or negative valence of the event in repeated exposure to a suspenseful film.

Since this study provides an analysis of physiological data, the problem of reverse inference should be addressed. As Poldrack (2006) pointed out, when a researcher infers human cognitive processing from physiological responses, the data should be analyzed and interpreted cautiously. Regarding SCL and HR, the physiological measures used in the current study, even though there are strong evidences that SC is closely related to arousal, HR is supposed to be associated with multiple cognitive and emotional processes, such as attention, information encoding, emotional arousal, and positive/negative emotional responses. Therefore, it should be noted that, although we used HR as a main index of attention, the interpretation of HR remains controversial.

From a broader and more general perspective, we emphasize the multidimensional aspect of the suspense experience, that is, the constituents of a suspenseful experience may span across different types of elements, such as the suspenseful narrative structure, the sympathy for likable characters, the evolutionary reaction toward threat and danger, and the musical tension from the soundtrack of the film. Audiences can feel suspense again due to musical tension, which can be experienced repeatedly (Koelsch et al., 2002). Another general explanation for the suspense paradox is that the response toward the suspenseful film might be related to the fast and instinctive reaction that belongs to the mode of “system 1” introduced in Kahneman’s (2011) theory. In such a case, we will experience suspense automatically and unintentionally when watching suspenseful scenes even repeatedly. In this way, the psychological impact of re-living suspense can be influenced by different dimensional factors. That is why we confront challenging and complicated problems when conducting research into suspense.

## Data Availability Statement

The raw data supporting the conclusions of this article will be made available by the authors, without undue reservation.

## Ethics Statement

The studies involving human participants were reviewed and approved by Institutional Review Board of Korea Advanced Institute of Science and Technology. The participants provided their informed consent to participate in this study.

## Author Contributions

CC, BP, and CS contributed to the conception and design of the study. CC and CS investigated the related work. CC and BP designed the experimental method, conducted the data processing, performed the statistical analysis, and revised the manuscript. CC conducted the experiment, collected the data, and wrote the draft of the manuscript. BP and CS provided the feedback. All authors contributed to the article and approved the submitted version.

## Conflict of Interest

The authors declare that the research was conducted in the absence of any commercial or financial relationships that could be construed as a potential conflict of interest.

## Footnotes

1 cf. The Oxford English Dictionary (1989) defines suspense as “a state of mental uncertainty, with expectation of or desire for decision, and usually some apprehension or anxiety as well as doubt (p. 320).”

## References

[B1] Bezdek M. A. Gerrig R. J. (2017). When narrative transportation narrows attention: changes in attentional focus during suspenseful film viewing. *Media Psychol.* 20 60–89. 10.1080/15213269.2015.1121830

[B2] Bolls P. D. Lang A. (2003). I saw it on the radio: the allocation of attention to high-imagery radio advertisements. *Media Psychol.* 5 33–55. 10.1207/S1532785XMEP0501_2

[B3] Bolls P. D. Lang A. Potter R. F. (2001). The effects of message valence and listener arousal on attention, memory, and facial muscular responses to radio advertisements. *Commun. Res.* 28 627–651. 10.1177/009365001028005003

[B4] Bradley M. M. (2009). Natural selective attention: orienting and emotion. *Psychophysiology* 46 1–11. 10.1111/j.1469-8986.2008.00702.x 18778317 PMC3645482

[B5] Bradley M. M. Codispoti M. Cuthbert B. N. Lang P. J. (2001). Emotion and motivation I: defensive and appetitive reactions in picture processing. *Emotion* 1 276–298. 10.1037/1528-3542.1.3.276 12934687

[B6] Bradley M. M. Lang P. J. (1994). Measuring emotion: the self-assessment manikin and the semantic differential. *J. Behav. Ther. Exp. Psychol.* 25 49–59. 10.1016/0005-7916(94)90063-9 7962581

[B7] Bradley M. M. Lang P. J. (2000). Affective reactions to acoustic stimuli. *Psychophysiology* 37 204–215. 10.1111/1469-8986.3720204 10731770

[B8] Brewer W. F. (1996). “The nature of narrative suspense and the problem of rereading,” in *Suspense: Conceptualizations, Theoretical Analyses, and Empirical Explorations*, eds Vorderer P. Wulff H. J. Friedrichsen M. (Mahwah, NJ: Lawrence Erlbaum Associates, Inc), 107–127. 10.1057/9780230106116_4

[B9] Brewer W. F. Lichtenstein E. H. (1982). Stories are to entertain: a structural-affect theory of stories. *J. Pragmat.* 6 473–486. 10.1016/0378-2166(82)90021-2

[B10] Busselle R. Bilandzic H. (2009). Measuring narrative engagement. *Media Psychol.* 12 321–347. 10.1080/15213260903287259

[B11] Cacioppo J. T. Tassinary L. G. (1990). “Psychophysiology and psychophysiological inference,” in *Principles of Psychophysiology: Physical, Social, and Inferential Elements*, eds Cacioppo J. T. Tassinary L. G. (New York, NY: Cambridge University Press), 3–33. 10.1017/9781107415782.001

[B12] Carroll N. (1996). “The paradox of suspense,” in *Suspense: Conceptualizations, Theoretical Analyses, and Empirical Explorations*, eds Vorderer P. Wulff H. J. Friedrichsen M. (Mahwah, NJ: Lawrence Erlbaum Associates, Inc), 71–91.

[B13] Carvalho S. Leite J. Galdo-Álvarez S. Gonçalves O. F. (2012). The emotional movie database (EMDB): a self-report and psychophysiological study. *Appl. Psychophysiol. Biofeedback* 37 279–294. 10.1007/s10484-012-9201-6 22767079

[B14] Chatman S. B. (1980). *Story and Discourse: Narrative Structure in Fiction and Film.* Ithaca, NY: Cornell University Press.

[B15] Codispoti M. De Cesarei A. Biondi S. Ferrari V. (2016). The fate of unattended stimuli and emotional habituation: behavioral interference and cortical changes. *Cogn. Affect. Behav. Neurosci.* 16 1063–1073. 10.3758/s13415-016-0453-0 27557884

[B16] Codispoti M. Ferrari V. Bradley M. M. (2006). Repetitive picture processing: autonomic and cortical correlates. *Brain Res.* 1068 213–220. 10.1016/j.brainres.2005.11.009 16403475

[B17] Codispoti M. Surcinelli P. Baldaro B. (2008). Watching emotional movies: affective reactions and gender differences. *Int. J. Psychophysiol.* 69 90–95. 10.1016/j.ijpsycho.2008.03.004 18433903

[B18] Comisky P. Bryant J. (1982). Factors involved in generating suspense. *Hum. Commun. Res.* 9 49–58. 10.1111/j.1468-2958.1982.tb00682.x

[B19] Fanti K. A. Vanman E. Henrich C. C. Avraamides M. N. (2009). Desensitization to media violence over a short period of time. *Aggress. Behav.* 35 179–187. 10.1002/ab.20295 19172659

[B20] Ferrari V. Mastria S. Codispoti M. (2020). The interplay between attention and long-term memory in affective habituation. *Psychophysiology* 57:e13572. 10.1111/psyp.13572 32239721

[B21] Gerrig R. J. (1989). Suspense in the absence of uncertainty. *J. Mem. Lang.* 28 633–648. 10.1016/0749-596x(89)90001-6

[B22] Gerrig R. J. (1993). *Experiencing Narrative Worlds: On the Psychological Activities of Reading.* New Haven, CT: Yale University Press.

[B23] Gerrig R. J. (1996). “Resiliency of suspense,” in *Suspense: Conceptualizations, Theoretical Analyses, and Empirical Explorations*, eds Vorderer P. Wulff H. J. Friedrichsen M. (Mahwah, NJ: Lawrence Erlbaum Associates, Inc), 93–105.

[B24] Gerrig R. J. (1997). Is there a paradox of suspense? A reply to Yanal. *Br. J. Aesthet.* 37 168–174. 10.1093/bjaesthetics/37.2.168

[B25] Grabe M. E. Lang A. Zhou S. Bolls P. D. (2000). Cognitive access to negatively arousing news: an experimental investigation of the knowledge gap. *Commun. Res.* 27 3–26. 10.1177/009365000027001001

[B26] Hoeken H. van Vliet M. (2000). Suspense, curiosity, and surprise: how discourse structure influences the affective and cognitive processing of a story. *Poetics* 27 277–286. 10.1016/s0304-422x(99)00021-2

[B27] Hubert W. de Jong-Meyer R. (1991). Autonomic, neuroendocrine, and subjective responses to emotion-inducing film stimuli. *Int. J. Psychophysiol.* 11 131–140. 10.1016/0167-8760(91)90005-I 1748588

[B28] Kahneman D. (2011). *Thinking, Fast and Slow.* New York, NY: Farrar, Straus and Giroux.

[B29] Knobloch S. Patzig G. Mende A.-M. Hastall M. (2004). Affective news: effects of discourse structure in narratives on suspense, curiosity, and enjoyment while reading news and novels. *Commun. Res.* 31 259–287. 10.1177/0093650203261517

[B30] Koelsch S. Schroger E. Gunter T. C. (2002). Music matters: preattentive musicality of the human brain. *Psychophysiology* 39 38–48. 10.1111/1469-8986.3910038 12206294

[B31] Lang A. Bolls P. Potter R. F. Kawahara K. (1999). The effects of production pacing and arousing content on the information processing of television messages. *J. Broadcast. Electron. Media* 43 451–475. 10.1080/08838159909364504

[B32] Lang A. Bradley S. D. Sparks J. V. Lee S. (2007). The motivation activation measure (MAM): how well does MAM predict individual differences in physiological indicators of appetitive and aversive activation? *Commun. Methods Meas.* 1 113–136. 10.1080/19312450701399370

[B33] Lang A. Chung Y. Lee S. Schwartz N. Shin M. (2005a). It’s an arousing, fast-paced kind of world: the effects of age and sensation seeking on the information processing of substance-abuse PSAs. *Media Psychol.* 7 421–454. 10.1207/S1532785XMEP0704_6 26627889

[B34] Lang A. Chung Y. Lee S. Zhao X. (2005b). It’s the product: do risky products compel attention and elicit arousal in media users? *Health Commun.* 17 283–300. 10.1207/s15327027hc1703_5 15855074

[B35] Lang A. Dhillon K. Dong Q. (1995). The effects of emotional arousal and valence on television viewers’ cognitive capacity and memory. *J. Broadcast. Electron. Media* 39 313–327. 10.1080/08838159509364309

[B36] Lehne M. Engel P. Rohrmeier M. Menninghaus W. Jacobs A. M. Koelsch S. (2015). Reading a suspenseful literary text activates brain areas related to social cognition and predictive inference. *PLoS One* 10:e0124550 10.1371/journal.pone.0124550 PMC4422438 25946306

[B37] Lehne M. Koelsch S. (2015). Toward a general psychological model of tension and suspense. *Front. Psychol.* 6:79. 10.3389/fpsyg.2015.00079 25717309 PMC4324075

[B38] Lim S. Reeves B. (2009). Being in the game: effects of avatar choice and point of view on psychophysiological responses during play. *Media Psychol.* 12 348–370. 10.1080/15213260903287242

[B39] Madrigal R. Bee C. Chen J. LaBarge M. (2011). The effect of suspense on enjoyment following a desirable outcome: the mediating role of relief. *Media Psychol.* 14 259–288. 10.1080/15213269.2011.596469

[B40] Park B. Bailey R. L. (2017). Application of information introduced to dynamic message processing and enjoyment. *J. Media Psychol.* 30 196–206. 10.1027/1864-1105/a000195

[B41] Poldrack R. A. (2006). Can cognitive processes be inferred from neuroimaging data? *Trends Cogn. Sci.* 10 59–63. 10.1016/j.tics.2005.12.004 16406760

[B42] Potter R. F. (2000). The effects of voice changes on orienting and immediate cognitive overload in radio listeners. *Media Psychol.* 2 147–177. 10.1207/S1532785XMEP0202_3

[B43] Potter R. F. Bolls P. (2012). *Psychophysiological Measurement and Meaning: Cognitive and Emotional Processing of Media.* New York, NY: Routledge.

[B44] Ravaja N. (2004). Contributions of psychophysiology to media research: review and recommendations. *Media Psychol.* 6 193–235. 10.1207/s1532785xmep0602_4

[B45] Ravaja N. Saari T. Salminen M. Laarni J. Kallinen K. (2006). Phasic emotional reactions to video game events: a psychophysiological investigation. *Media Psychol.* 8 343–367. 10.1207/s1532785xmep0804_2

[B46] Reeves B. Lang A. Kim E. Y. Tatar D. (1999). The effects of screen size and message content on attention and arousal. *Media Psychol.* 1 49–67. 10.1207/s1532785xmep0101_4

[B47] Rottenberg J. Ray R. D. Gross J. J. (2007). “Emotion elicitation using films,” in *The Handbook of Emotion Elicitation and Assessment*, eds Coan J. A. Allen J. J. B. (New York, NY: Oxford University Press), 9–28.

[B48] Schmälzle R. Grall C. (2020). The coupled brains of captivated audiences: an investigation of the collective brain dynamics of an audience watching a suspenseful film. *J. Media Psychol.* 10.1027/1864-1105/a000271

[B49] Skulsky H. (1980). On being moved by fiction. *J. Aesthet. Art Crit.* 39 5–14. 10.2307/429914

[B50] Smith N. K. Larsen J. T. Chartrand T. L. Cacioppo J. T. Katafiasz H. A. Moran K. E. (2006). Being bad isn’t always good: affective context moderates the attention bias toward negative information. *J. Pers. Soc. Psychol.* 90 210–220. 10.1037/0022-3514.90.2.210 16536647

[B51] Suckfull M. (2000). Film analysis and psychophysiology effects of moments of impact and protagonists. *Media Psychol.* 2 269–301. 10.1207/S1532785XMEP0203_4

[B52] Sundar S. S. Kalyanaraman S. (2004). Arousal, memory, and impression-formation effects of animation speed in web advertising. *J. Advert.* 33 7–17. 10.1080/00913367.2004.10639152

[B53] Tan E. Diteweg G. (1996). “Suspense, predictive inference, and emotion in film viewing,” in *Suspense: Conceptualizations, Theoretical Analyses, and Empirical Explorations*, eds Vorderer P. Wulff H. J. Friedrichsen M. (Mahwah, NJ: Lawrence Erlbaum Associates, Inc), 149–188.

[B54] Turner J. R. (1994). *Cardiovascular Reactivity and Stress: Patterns of Physiological Response.* New York, NY: Springer Science & Business Media.

[B55] Vorderer P. (1996). “Toward a psychological theory of suspense,” in *Suspense: Conceptualizations, Theoretical Analyses, and Empirical Explorations*, eds Vorderer P. Wulff H. J. Friedrichsen M. (Mahwah, NJ: Lawrence Erlbaum Associates, Inc), 232–254.

[B56] Walton K. L. (1978). Fearing fictions. *J. Philos.* 75 5–27. 10.2307/2025831

[B57] Walton K. L. (1990). *Mimesis as Make-Believe: On the Foundations of the Representational Arts.* Cambridge, MA: Harvard University Press.

[B58] Wixted J. T. Ebbesen E. B. (1991). On the form of forgetting. *Psychol. Sci.* 2 409–415. 10.1111/j.1467-9280.1991.tb00175.x

[B59] Yanal R. J. (1996). The paradox of suspense. *Br. J. Aesthet.* 36 146–158. 10.1093/bjaesthetics/36.2.146

[B60] Zillmann D. (1991a). “Empathy: affect from bearing witness to the emotions of others,” in *Responding to the Screen: Reception and Reaction Processes*, eds Bryant J. Zillmann D. (Mahwah, NJ: Lawrence Erlbaum Associates, Inc), 135–167.

[B61] Zillmann D. (1991b). “The logic of suspense and mystery,” in *Responding to the Screen: Reception and Reaction Processes*, eds Bryant J. Zillmann D. (Mahwah, NJ: Lawrence Erlbaum Associates, Inc), 281–303.

[B62] Zillmann D. (1995). Mechanisms of emotional involvement with drama. *Poetics* 23 33–51. 10.1016/0304-422X(94)00020-7

[B63] Zillmann D. (1996). “Psychology of suspense in dramatic exposition,” in *Suspense: Conceptualizations, Theoretical Analyses, and Empirical Explorations*, eds Vorderer P. Wulff H. J. Friedrichsen M. (Mahwah, NJ: Lawrence Erlbaum Associates, Inc), 199–231.

[B64] Zillmann D. Hay T. A. Bryant J. (1975). The effect of suspense and its resolution on the appreciation of dramatic presentations. *J. Res. Pers.* 9 307–323. 10.1016/0092-6566(75)90005-7

